# Staple Rivet Teeth

**Published:** 1847-10

**Authors:** 


### "*Staple Rivet Teeth.*"

We have recently made an important improvement in our plate teeth, by which the liability of the rivets to draw from the teeth is prevented. It consists in introducing the two rivets into a tooth, in the form of a staple. We have also made some other improvements in the manufacture of mineral teeth, whereby their resemblance to natural teeth is rendered so close, as almost to defy detection, when placed in the mouth by a skilful practitioner.

We have a very large and full assortment on hand, at our establishments in Philadelphia and New York, which will enable us to supply orders of any size, on the shortest notice.

The above we take from Stockton, & Co., Dental Register. We are glad to see that an improvement, which has been so long needed, has at length made its appearance. As Dentists, we naturally look to the manufacturer for improvements in this department. We hope that this spirit of improvement may prevail, until the body shall be made so perfect, that it will hold together as tenaciously as these "Staple Rivets" retain their position in the teeth. We have used some of the above manufacture, and, as yet, have not been mortified by having plates returned with teeth broken off—nothing is more annoying than the frequency with which many of our best looking teeth break or draw from the rivets which hold them to the plate. It matters not how well the dentist may have performed his work, he must stand responsible to his patient. The price of a tooth is nothing compared to the time consumed, the labor and the loss incident to the repair of such jobs.

We make not these remarks intending to reflect any discredit on teeth from the establishment named, for we have found them fully equal to any we have used. Yet we wish to draw the attention of manufacturers to the fact, that teeth are not made to perfection as yet—true, compared with those used fifteen years ago and they appear perfect.

Yet, in two or three particulars, improvements may be made: 1st. The palital surface of the incissors and canine have not as much of the shape of the natural organs, as is desired to restore perfect enunciation. This is applicable also to the bicuspides and molares. The labial surface, although not always perfect, yet generally is much more so than the palital.

2d. The strength or tenacity of the body is not generally such as it should be. This may, and often does depend on the preparation of the materials of which the body is composed. A coarse body will not make good teeth. Introduce, gentlemen, as many labor saving machines as you please, but fail not to employ sufficient labor to make as perfect as possible these important specimens of your skill. We hope, next year, to see the Mississippi Valley Association of Dental Surgeons offer a premium for the best formed and the most perfect teeth which may be presented at their next meeting.

We would remind Messrs. Stockton & Co., that they have an extensive *establishment* for the sale of their teeth in this city, and, that if they intend to retain the *immense amount* of business done at this depot for their benefit, it will be necessary to forward a large supply of their new teeth to our friend, on the corner of Fourth and Walnut.

Dentists generally of this city, (I might say of this great Valley,) feel interested in sustaining such an establishment. They cannot do this by sending orders to the east. Many of us feel that it would be doing injustice to the accommodating agent, whom we have induced to embark in this business. In conclusion we would say, that we feel anxious to see "the other important improvements," referred to in the article which has drawn out these remarks. Dr. Brown, at the Melodeon corner, will, we know, give us timely warning of their arrival.—CIN ED.

NOTE.—Since writing the above, we have been shown, by Dr. Brown, a lot of these teeth, just received from the manufacture, and we have no doubt they will soon be disposed of, at the small additional cost of one cent per tooth.—CIN. ED.

